# Spindle Cell Rhabdomyosarcoma of the Inguinal Region Mimicking a Complicated Hernia in the Adult—An Unexpected Finding

**DOI:** 10.3390/medicina59091515

**Published:** 2023-08-23

**Authors:** Valentin Titus Grigorean, Radu Serescu, Andrei Anica, Violeta Elena Coman, Ştefan Iulian Bedereag, Roxana Corina Sfetea, Mircea Liţescu, Iancu Emil Pleşea, Costin George Florea, Cosmin Burleanu, Anwar Erchid, Ionuţ Simion Coman

**Affiliations:** 1 Discipline of General Surgery, “Bagdasar-Arseni” Clinical Emergency Hospital, 10th Clinical Department—General Surgery, Faculty of Medicine, “Carol Davila” University of Medicine and Pharmacy, 37 Dionisie Lupu Street, 020021 Bucharest, Romania; valentin.grigorean@umfcd.ro (V.T.G.); elena.coman@umfcd.ro (V.E.C.); ionut.coman@umfcd.ro (I.S.C.); 2 General Surgery Department, “Bagdasar-Arseni” Clinical Emergency Hospital, 12 Berceni Road, 041915 Bucharest, Romania; costinflorea1990@gmail.com (C.G.F.); burleanucosmin@gmail.com (C.B.); erchid.anwar@yahoo.com (A.E.); 3 Amethyst Medical Center, 42 Odăii Street, 075100 Otopeni, Romania; raduserescu@gmail.com (R.S.); dr.andrei.anica@gmail.com (A.A.); 4 Ph.D. School, “Carol Davila” University of Medicine and Pharmacy, 37 Dionisie Lupu Street, 020021 Bucharest, Romania; 5 Pathology Department, “Bagdasar-Arseni” Clinical Emergency Hospital, 12 Berceni Road, 041915 Bucharest, Romania; stefanbedereag@gmail.com (Ş.I.B.); pie1956@yahoo.com (I.E.P.); 6 Discipline of Modern Languages, 3rd Preclinical Department—Complementary Sciences, Faculty of Medicine, “Carol Davila” University of Medicine and Pharmacy, 37 Dionisie Lupu Street, 020021 Bucharest, Romania; roxana.sfetea@umfcd.ro; 7 Discipline of Surgery and General Anesthesia,“Sf. Ioan” Clinical Emergency Hospital, 2nd Department, Faculty of Dental Medicine, “Carol Davila” University of Medicine and Pharmacy, 37 Dionisie Lupu Street, 020021 Bucharest, Romania; 8 General Surgery Department, “Sf. Ioan” Clinical Emergency Hospital, 13 Vitan-Bârzeşti Road, 042122 Bucharest, Romania

**Keywords:** rhabdomyosarcoma, spindle cell, inguinal, hernia, adult

## Abstract

Rhabdomyosarcoma is a rare tumor that is diagnosed mostly in children and adolescents, rarely in adults, representing 2–5% of all soft tissue sarcomas. It has four subtypes that are recognized: embryonal (50%), alveolar (20%), pleomorphic (20%), and spindle cell/sclerosing (10%). The diagnosis of rhabdomyosarcoma is based on the histological detection of rhabdomyoblasts and the expression of muscle-related biomarkers. Spindle cell/sclerosing rhabdomyosarcoma consists morphologically of fusiform cells with vesicular chromatin arranged in a storiform pattern or long fascicles, with occasional rhabdomyoblasts. Also, dense, collagenous, sclerotic stroma may be seen more commonly in adults. We present a rare case of an adult who presented to the hospital with a tumor in the left inguinal area, was first diagnosed with a left strangulated inguinal hernia and was operated on as an emergency, although the diagnosis was ultimately a spindle cell rhabdomyosarcoma of the inguinal region.

## 1. Introduction

Rhabdomyosarcoma is defined as a malignant mesenchymal tumor that has skeletal muscle differentiation, with four subtypes that are recognized: embryonal (50%), alveolar (20%), pleomorphic (20%), and spindle cell/sclerosing (10%) [1]. A more detailed classification includes the botryoid rhabdomyosarcoma, which is a subtype of the embryonal rhabdomyosarcoma, characteristic of the mucosal surfaces on the walls of hollow organs, such as the vagina, the uterine cervix, the bladder, the biliary tract, or the nasopharynx of infants [2,3]. 

Rhabdomyosarcoma is a rare tumor that is diagnosed mostly in children and adolescents, but rarely in adults, representing 2–5% of all soft tissue sarcomas [4]. Around two-thirds of the cases occur in children younger than 6 years of age [5]. Rhabdomyosarcomas account for 3% of childhood cancers and 2% of adolescent cancers and its incidence makes information regarding its characteristics very limited [6,7,8]. 

The diagnosis of rhabdomyosarcoma is based on the histological detection of rhabdomyoblasts and the expression of muscle-related biomarkers. Despite showing elements of skeletal muscle differentiation, rhabdomyosarcomas can develop in areas where skeletal muscle is absent [9]. 

Following the efforts of the Intergroup Rhabdomyosarcoma Study, Newton and al. proposed a classification for the prediction of the outcome of patients with various types of rhabdomyosarcomas (Table 1) [10,11]: 

Cavazzana et al. first described spindle cell rhabdomyosarcomas in children [12], while Rubin et al. published the first occurrence of spindle cell rhabdomyosarcomas in adults [13]. Furthermore, Mentzel et al. reported sclerosing pseudovascular rhabdomyosarcoma, an additional morphological variant of spindle cell rhabdomyosarcoma, in adults [14]. Although initial classifications included the spindle cell/sclerosing rhabdomyosarcoma as a subtype of embryonal rhabdomyosarcoma [15], it was classified as a separate entity by the World Health Organization in 2013 [16]. Morphologically, it consists of fusiform cells with vesicular chromatin arranged in a storiform pattern or long fascicles, with occasional rhabdomyoblasts. Also, dense, collagenous, sclerotic stroma may be seen more commonly in adults [1]. 

We present a rare case of an adult who presented to the hospital with a tumor in the left inguinal area, was first diagnosed with a left strangulated inguinal hernia and was operated on as an emergency before diagnosis with a spindle cell rhabdomyosarcoma of the inguinal region.

## 2. Detailed Case Description

A 69-year-old male patient, known to have type I obesity, arterial hypertension, and type II diabetes, for which he takes oral medication, presents to the Emergency Department of our hospital with a tumor in the left inguinal region. The patient reports that he observed the tumor several months ago, during which it grew and was accompanied by local pain. 

The local clinical exam reveals, in the left inguinal region, a mass of approximately 8/5 cm (cm), that has an increased consistency, adherent to the superficial and profound planes. Given its location, the pain reported by the patient, and our inability to mobilize the tumor, we establish an initial diagnosis of a strangulated left inguinal hernia.

A chest X-ray exam reveals right lateral-basal right pleurisy in small quantity, while the abdominal X-ray exam does not reveal pneumoperitoneum or air-fluid levels. Biologically, we find hyperglycemia, with a value of 170 milligrams (mg)/deciliter (dL).

We perform an emergency surgical procedure using a left inguinal approach. We identify a tumoral mass in the inguinal canal, which encompasses the spermatic cord up to the profound inguinal orifice and modifies the local anatomy, making it difficult to identify the normal structures of this area. We are not able to identify a hernia sac, but the certain exclusion of a strangulated hernia is only made after we perform an additional midline incision in the lower abdomen, with the endo-abdominal inspection of the inguinofemoral region. The dissection in the inguinal area is difficult due to the infiltrative characteristic of the tumor, which also invades the inguinal ligament and is in close vicinity to the external iliac vessels and the left pubic ramus. Due to the lack of an extemporaneous histopathological analysis and the impossibility of performing a complete resection of the tumor without the sacrifice of the left testicle, we limit our procedure to a biopsy from the tumor.

Subsequently, the patient has a favorable surgical evolution, being discharged on the fifth postoperative day. 

The histopathological examination of the biopsy specimen reveals adipose and muscle tissue that includes a diffuse infiltrative mesenchymal tumoral proliferation, with a fasciculated pattern made by elongated cells, with obvious pleomorphism. Associated with these elements, a component of multinucleated cells with eosinophilic cytoplasm, of the rhabdoid type (3 mitoses/high-power-field (HPF)) is observed. 

Given the histopathologic result, suggesting a rhabdomyosarcoma, the patient receives an oncologic evaluation, including one from an expert based in an European Society for Medical Oncology (ESMO) Sarcoma reference center. The recommendation, taking into account the age and the associated diseases of the patient, is for a surgical procedure with microscopic complete resection (R_0_) if possible, followed by adjuvant radiotherapy and adjuvant chemotherapy with Doxorubicin.

**Figure 1:**
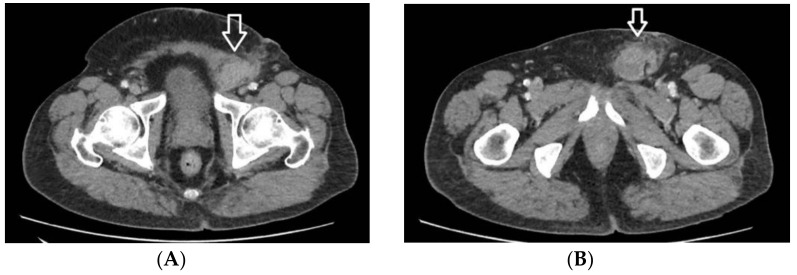
Abdominal CT image of the tumor (marked with white arrow) at different levels (**A**,**B**).

The patient returns to our department 10 days after the first discharge. A thoracic, abdominal, and pelvic computer tom ography (CT) is performed, revealing a nodular tumor with left inguinal topography, imprecisely delimited outline, iodophil, dimensions of 51/33/73 mm (transverse/anteroposterior/craniocaudal diameters). The lesion is not distinguishable from the content of the inguinal canal and presents relations posteriorly and laterally with the external iliac vessels, with a fatty cleavage plane to them, superiorly and medially with the inferior epigastric vessels, without cleavage plane, invasive medially in the rectus abdominis muscle, superior and laterally invasive in the transversus abdominis muscle, and posteriorly and inferiorly in contact with the pectineus muscle. In addition to these relations the CT reveals a small lymph node of 15/10 mm between the portal vein and inferior cava vein. It also reveals multiple thyroid nodules and a pseudo-nodular pulmonary consolidation, with subpleural topography in the posterobasal segment of the right inferior pulmonary lobe, most probably of atelectatic origin (Figure 1A,B).

**Figure 2:**
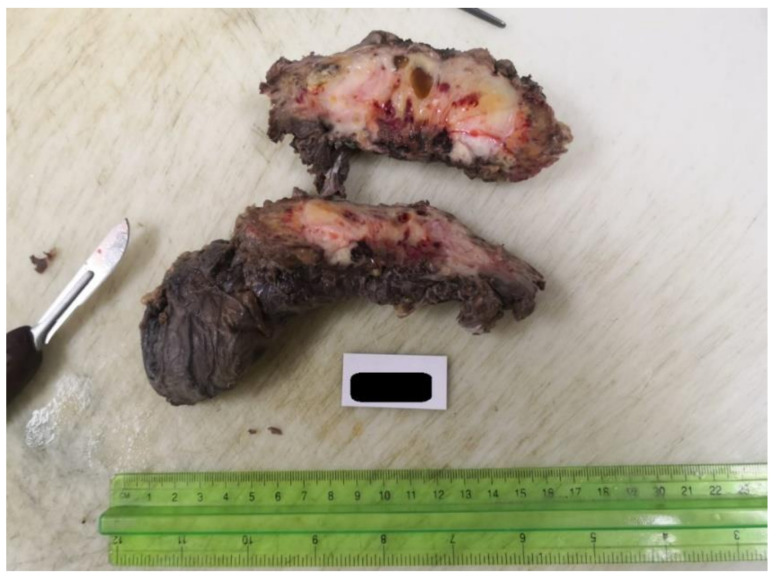
The resected tumor en bloc with the left testicle.

We perform the surgical procedure, in which we manage to resect the entire tumor en bloc with the spermatic cord and left testicle, including its invasion in the muscles, the invasion of the pubic ramus with resection of the periosteum and the dissection from the left external iliac vessels (Figure 2). Subsequently, the patient has a favorable surgical evolution, being discharged on the ninth postoperative day. 

**Figure 3:**
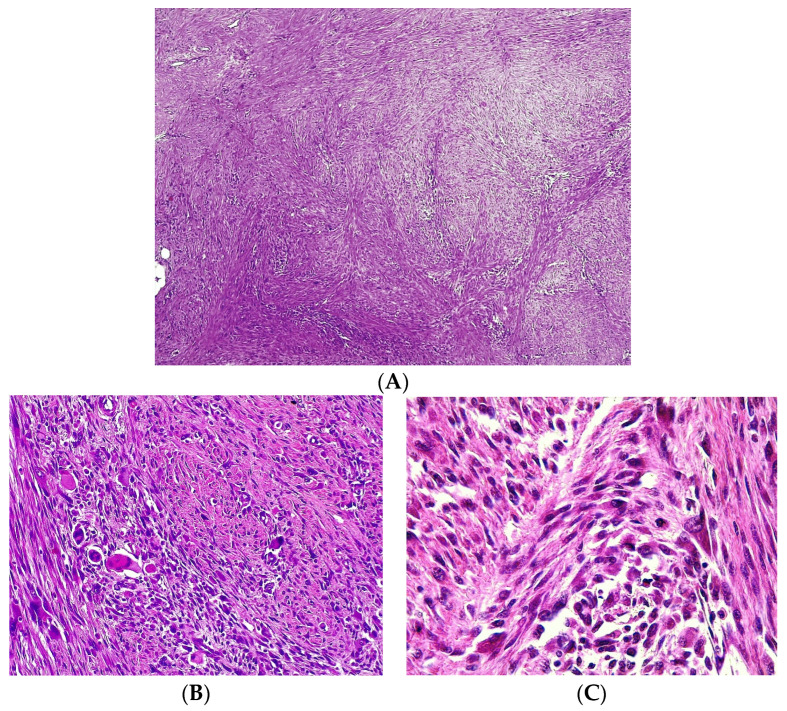
The histopathologic aspect of the specimen—optic microscopy, hematoxylin-eosin staining, magnifying of 4× (**A**), 20× (**B**), and 40× (**C**), respectively.

The histopathological examination of the resected tumor reveals a spermatic cord, with the architecture disrupted by malignant mesenchymal tumor proliferation. This has a nodular, fascicular pattern, in the form of intersecting, confluent bundles, forming extensive groups of spindle-shaped tumor cells and frequent tumor cells with abundant cytoplasm, intensely eosinophilic, with nuclei dislocated at the periphery of the cell having a rhabdoid appearance. In addition, some multinucleated giants can be seen, with marked cytonuclear atypia, relatively rare mitotic figures, and the presence of atypical mitoses (approximately 5 mitoses/10 HPF), narrow foci of tumor necrosis (<50% of the examined tumor mass), areas of hemorrhage, siderophagia and polymorphic inflammatory infiltrate with a diffuse intratumoral and peritumoral disposition. In addition, perineural invasion (PNI+) and lymphovascular invasion (LVI+) are present (Figure 3A–C). The proximal limit of resection is free, and the left testicle and epididymis are not invaded by the tumor. 

The histopathological analysis and the immunohistochemistry tests (Table 2) reveal a malignant paratesticular mesenchymal proliferation, compatible with a spindle cell rhabdomyosarcoma.

The patient is evaluated again by the oncologist who recommends a cardiology evaluation before starting the adjuvant therapy. The patient receives a transthoracic echocardiogram, revealing a normal global ejection fraction, normal aspect of both ventricles with preserved systolic function, and only mild diastolic dysfunction, but with normal filling pressures. 

**Figure 4:**
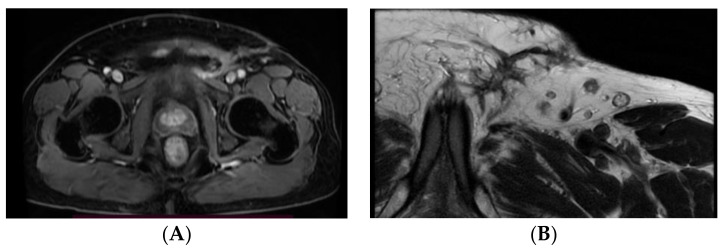
Postoperative MRI aspect of the left inguinal area—axial sections at different levels showing inflammatory modifications (**A**) and several lymph nodes (**B**).

About one and a half months after the second surgical procedure, pelvic Magnetic Resonance Imaging (MRI) is performed (Figure 4A,B), revealing a recent postoperative aspect in the left inguinal and femoral area, with associated inflammatory modifications. In addition, it reveals external iliac lymph nodes of a maximum 21/7 mm size on the right side and 19/6 mm on the left side, and inguinofemoral lymph nodes of a maximum of 13/8 mm in size on the right side and 13/9 mm on the left side. In addition, the pectineal muscle presents in its 1/3 anterior region a few muscle bundles with modified signal (short tau inversion recovery (STIR) hypersignal), gadolinium enhancement—inflammatory modifications or tumor invasion, as well as a few satellite lymph nodes of maximum 7/6 mm in size.

**Figure 5:**
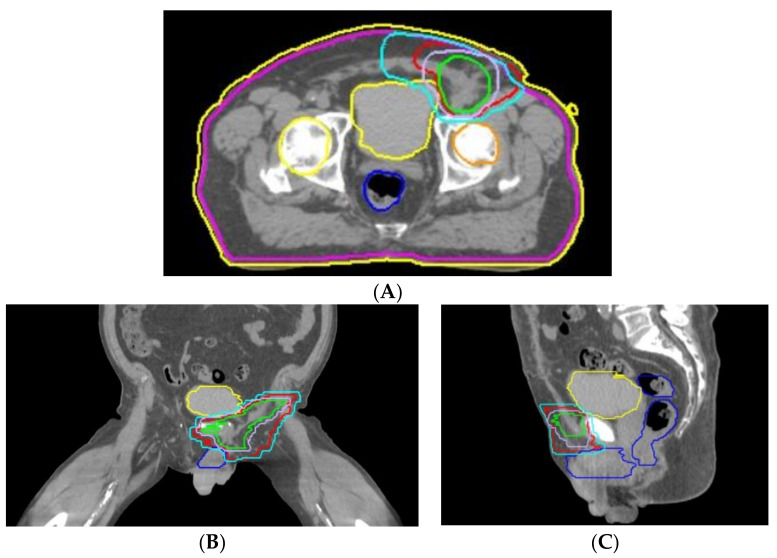
Sections of external radiation areas: clinical target volume (CTV) 1—red (50 Gy); PTV1—dark blue (50 Gy); CTV2—green (66 Gy); PTV2—purple (66 Gy); axial (**A**), coronal (**B**) and sagittal (**C**) sections. Sky blue, pink, yellow and orange have no clinical significance in this case.

Shortly after the MRI exam, the patient undergoes radiotherapy for a month and a half, with external radiation in two areas at a planned target volume (PTV): left inguinal region (PTV1) and tumor bed (PTV2). The total dose for PTV1 was 50 Grays (Gy) (25 fractions of 2 Gy, 5 days a week, conventional fractionation), using arc therapy with volume modulation of intensity (IMRT-VMAT technique). The total dose for PTV2 was 66 Gy (33 fractions of 2 Gy, 5 days a week, conventional technique), using the IMRT-VMAT technique (Figure 5A–C).

**Figure 6:**
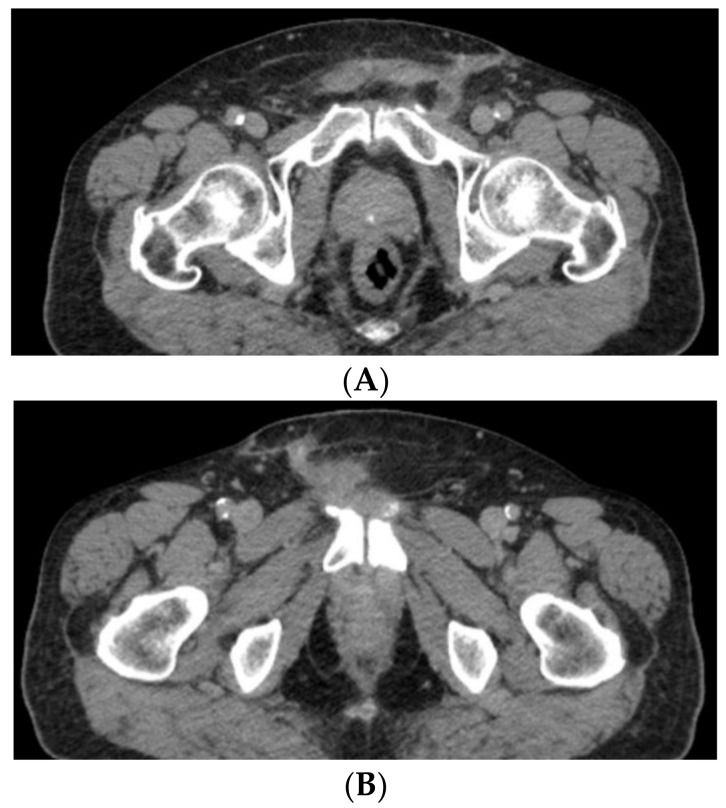
CT scan aspect, over three months after the tumor resection, at various levels, axial sections (**A**,**B**).

A week after the completion of the radiotherapy, a CT scan of the thorax, abdomen and pelvis is performed, showing no suggestive CT signs for local left inguinal tumoral recurrence and no lumbar-aortic or pelvic suspect lymph nodes (Figure 6A,B).

After the insertion of a port-a-cath, 5 months after the resection of the tumor, the patient undergoes six sessions of chemotherapy with Doxorubicin 60 mg/m^2^, one dose every 3 weeks. 

**Figure 7:**
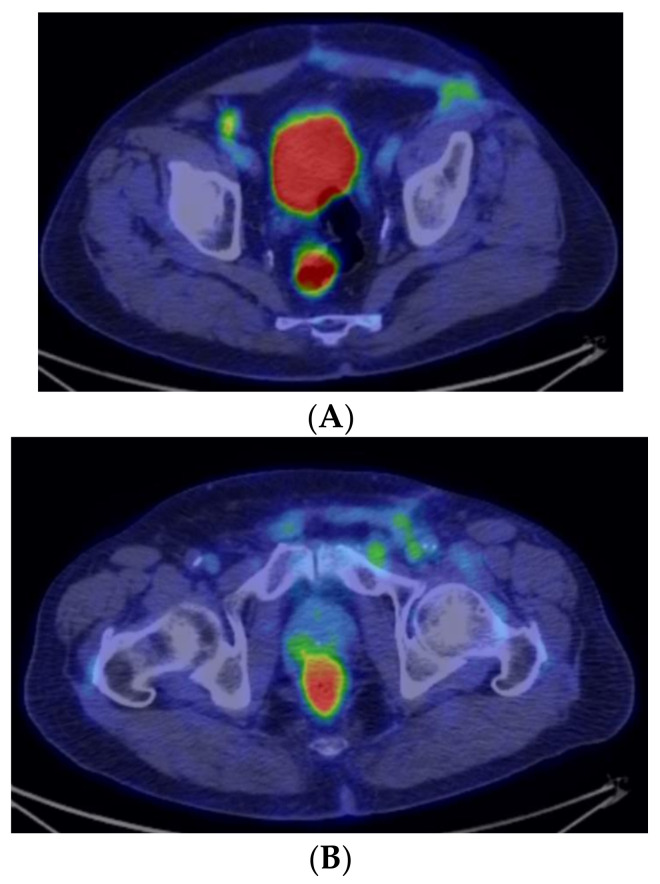
Postoperative PET-CT aspect, at different levels—axial sections (**A**,**B**).

Interspersed with the chemotherapy sessions, 6 months after the surgical procedure, the patient undergoes a positronic emission tomography (PET-CT).This reveals: a few lymph nodes with low uptake for fluorodeoxyglucose (F18-FDG) located on the right side of the trachea (not suggesting oncological lesions), encapsulated pleural fluid with pachypleuritis and atelectasis, diffuse enhanced uptake of the mentioned substance in the colon in the context of the oral antidiabetic treatment, and left inguinal modifications, diffuse and with minimal uptake. The conclusion of the PET-CT is that there are no active metabolic lesions with oncologic interest (Figure 7A,B). 

After the chemotherapy, two more thoracic, abdominal, and pelvic CT scans are performed, as well as a head CT scan, with no evidence of tumor relapse or metastases, with the most recent being made just a few days before the submission of this research. 

At present, 15 and a half months after the resection of the rhabdomyosarcoma, the patient is in good condition, monitored closely, and presenting with an incisional hernia at the site of the abdominal midline incision, which we will repair after the oncological assessment. 

## 3. Discussion

Spindle cell/sclerosing rhabdomyosarcoma represents 3–10% of rhabdomyosarcomas, affecting infants, children, and adults (ICD-O coding—8912/3) [17]. Studies show a strong predilection for young patients, with a mean age of around 7 years [15,18]. It affects both sexes, but some studies report a male preponderance with a ratio of 6:1 [19,20], while the presence of the MYOD1-mutant genetic subtype reveals a decreased male-to-female ratio [21]. 

In contrast to the spindle cell rhabdomyosarcoma in children and adolescents, where the most commonly affected region is the genitourinary tract and the orbital area, the main location of the spindle cell rhabdomyosarcoma in adults is in the head and neck region (except the orbital area) and the deep soft tissue of the extremities [22,23,24,25]. In addition, although they have a better prognosis compared to other subtypes of rhabdomyosarcoma, spindle cell rhabdomyosarcoma tumors are very aggressive and associated with a poor prognosis in adults, compared to this subtype of tumors in children [14,26]. 

The particularity of our patient was the unusual age of discovery for this subtype of rhabdomyosarcoma and the location of the tumor, mimicking a strangulated inguinal hernia that forced us to perform an emergency surgical procedure at that moment. 

Regarding the pathogenesis of spindle cell rhabdomyosarcoma, several groups have been described:

- Spindle cell/sclerosing rhabdomyosarcoma with a somatic activating mutation of the MYOD1 gene at position Lys122 that can be homo- or heterozygous. The mutated gene interacts with the MYC oncogene. This subtype can be encountered both in children in adults, being characterized by a poor prognosis, especially in children and adolescents, with the latter group age presenting a characteristic mutation of MyoD1 p.Leu122Arg [21,27,28];
- Variants with a rearrangement in the VGLL2/NCOA2 genes, that occur in children under the age of five or as congenital neoplasms. The prognosis is good, but with a high risk of local recurrence [16,29];
- Patients with spindle cell rhabdomyosarcoma developing in their bones, with EWSR1/FUS-TFCP2 or MEIS1-NCOA2 translocations. The presence of these mutations in adults has a very poor prognosis [30,31,32,33,34,35];
- Cases that do not have the alterations described above, encountered most commonly around the area of the testis or within the abdominal cavity [36];
- Patients with spindle cell rhabdomyosarcoma without molecular alterations [27].

Spindle cell/sclerosing rhabdomyosarcoma appears as a variably circumscribed tumor, with a size ranging between 1.5 and 35 cm. It shows a white-to-tan surface with a whorled appearance. Necrosis and cystic degeneration can also be present [17]. 

Histopathologically, spindle cell rhabdomyosarcoma is identified by cellular fascicles in spindle cells, with an intersecting or herringbone growth pattern, similar to fibrosarcoma or leiomyosarcoma. The spindled cells have pale eosinophilic cytoplasm and blunted, fusiform or ovoid, centrally located nuclei with small undistinguished nucleoli. Hyperchromatic nuclei, mitotic figures, and nuclear atypia are often present. In addition, primitive undifferentiated areas with round cells and hyperchromatic nuclei can also be present focally. Tadpole or strap cells, rhabdomyoblasts with elongated eosinophilic tails with cross-striations, can sometimes be identified [17]. Sclerosing rhabdomyosarcomas have prominent sclerosis/hyalinization, with tumor cells in various arrangements:cords, nests, microalveoli, or trabeculae in pseudovascular growth pattern. Areas with sclerosis may mimic osteosarcoma due to the extensive formation of the matrix [12,14,21,23,36]. Intraosseous spindle cell rhabdomyosarcoma, described recently, presents, apart from the typical spindle cell morphology, as areas of distinctly epithelioid cells arranged in fascicles and sheets [29,30,33]. 

Immunohistochemically, spindle cell/sclerosing rhabdomyosarcoma is characterized by the diffuse expression of desmin in all cases, with only the focal expression of myogenin (Myf-4) in most cases. MyoD1 staining can be diffuse or focal in the spindle cell tumors, but it is usually present in a diffuse pattern in sclerosing cases. Staining for smooth muscle actin (SMA) and muscle-specific actin (MSA) is usually absent. In addition, a positive result in cytokeratin and anaplastic lymphoma kinase (ALK) can be seen in the intraosseous spindle cell rhabdomyosarcoma [17]. 

According to the acknowledged histopathological description of spindle cell rhabdomyosarcoma, histopathological examination of the resected specimen in our patient revealed extensive groups of spindle-shaped tumor cells and frequent tumor cells with eosinophilic cytoplasm, with nuclei of rhabdoid appearance. At the immunohistochemical exam: desmin was focally positive, MyoD1 was diffuse positive, myogenin was focally positive, and, atypically, actin was positive in the tumoral proliferation. Furthermore, vimentin was diffuse and intensely positive, while Ki67 was positive in approximately 25% of the tumoral cells in our patient. Various published cases in the literature have shown variable values of Ki67 expression. Kacar et al. presented a case of spindle cell rhabdomyosarcoma with a Ki67 proliferation index < 2% [37], Jakkampudi et al. published a case with positivity of 40–50% for Ki67 [19] and Zhao et al. described a series of cases diagnosed with spindle cell/sclerosing rhabdomyosarcoma in which the proliferative index Ki67 varied from 15% to 80% [38]. The markers EMA and CD34 were negative in tumoral proliferation. Also, characteristically, spindle cell rhabdomyosarcoma has a negative expression for caldesmon, S-100 protein or glial fibrillary acidic protein (GFAP) [39], but the immunohistochemical examination for our patient did not include these markers. 

Rhabdomyosarcoma should be treated by a multidisciplinary team. Surgery is the basic therapeutic option for these patients, regardless of the risk group to which they belong. It should first be considered after diagnosis with the intent of complete resection of the tumor and obtaining microscopically radical surgical margins—R_0_ [27,40]. If metastases to lymph nodes are present, radical radiotherapy is used, but given the complications of radiotherapy, a histopathological examination of the lymph nodes should be made to exclude a reactive, non-neoplastic lymphadenopathy. Sparring treatment is usually preferred in case of the involvement of extremities. 

In the case of our patient, we resected the recommended 5 cm of normal tissue cranially to the tumor and the histopathological result revealed a tumor-free limit of resection. Distally, we were forced to perform a left orchiectomy, with the histopathological analysis revealing no invasion of the left testicle and epididymis. Circumferentially, the limit of 5 cm could not be achieved because of the particular area involved, the inguinal canal, but we managed to perform a complete resection of the tumor after dissecting it from the external iliac vessels and a resection of the pubic ramus periosteum. 

Radical radiotherapy should be considered in the case of extensive local tumoral invasion without the possibility of radical surgery, or in the case of other contraindications for surgical treatment [4,27,41]. Depending on the localization and clinical group (radical radiotherapy or supplementary radiotherapy after the surgical procedure), the total doses for radiotherapy vary between 50 and 65 Gy, with individual, personalized regiments for each patient, also taking into account the age of the patient [42,43].

Regarding chemotherapy, adding neoadjuvant and adjuvant therapy to the patients with metastases obtained a from 60% to 90% 5-year survival. In patients over 16 years of age and adults, the results were determined to be worse, with a 5-year survival of only 30–40% [27]. Various chemotherapy drugs are used, either in monotherapy or in association with two or three drugs: Vincristine, Doxorubicin, Ifosfamide, Dactinomycin, Cyclophosphamide, Actinomycin, and Etoposide. The subject of adjuvant and neoadjuvant radiotherapy and chemotherapy is one of actuality and several research studies are trying to determine the optimal protocols for the treatment of these tumors [36,44,45,46,47,48].

After finishing the oncological treatment, the patient should be observed carefully. The recommended procedure involves a physical examination and imaging studies such as CT and MRI of the primary localization, and CT of the chest, abdomen, and pelvis. Medical evaluation should take place every three months for the first two years, then every six months for the next three years, and subsequently once a year [27]. 

The most important factors for predicting the overall survival in patients with spindle cell rhabdomyosarcoma are the presence of metastatic disease at the first evaluation, tumor size, obtaining a negative margin status after primary surgical resection, and the response to chemotherapy [22,24,44].

In our case, after the complete surgical resection of the tumor, the patient responded well to radiotherapy and chemotherapy, whilst being closely monitored clinically and imagistically by multiple CT scans of the chest, abdomen, and pelvis, a pelvic MRI, and a PET-CT scan. These images showed no tumor relapse up until this moment, 15 months after the surgery. 

## 4. Conclusions

Spindle cell rhabdomyosarcoma is a rare form of malignant mesenchymal tumor that occurs especially often in children and adolescents. In adults, especially the elderly, the presence of this type of tumor in the inguinal area is very uncommon and may come as a surprise, due to its resemblance to a complicated inguinal hernia. 

The management of these cases is complex and involves the complete surgical resection of the tumor when possible, and associated radiotherapy and chemotherapy, as well as a strict follow-up, with frequent clinical and imagistic evaluations. 

## Figures and Tables

**Table 1:** Prognosis of various types of rhabdomyosarcoma.

Prognosis	Type
Superior prognosis	Botryoid rhabdomyosarcoma
	Spindle cell rhabdomyosarcoma
Intermediate prognosis	Embryonal rhabdomyosarcoma
Poor prognosis	Alveolar rhabdomyosarcoma
	Undifferentiated rhabdomyosarcoma

**Table 2:** Results of the immunohistochemistry exam.

Immunohistochemistry Marker	Result
Vimentin	Diffuse and intense positive in the tumoral proliferation
Myogenic determination gene (MyoD1)	Diffuse positive in the tumoral proliferation
Myogenin (Myf-4)	Focal positive in the tumoral proliferation
Desmin	Focal positive in the tumoral proliferation
Actin	Positive in the tumoral proliferation
Epithelial membrane antigen (EMA)	Negative in the tumoral proliferation
CD34	Negative in the tumoral proliferation; the presence of internal control
Ki67	Positive in approximately 25% of the tumoral cells

## Data Availability

Data is unavailable due to privacy or ethical restrictions.
